# Low Temperature Plasma Impairs the Pathogenicity of *Alternaria* sp. by Disrupting Its Cell Membrane and Redox Homeostasis

**DOI:** 10.3390/foods15152702

**Published:** 2026-07-31

**Authors:** Xinqi Zhang, Yaqing Fan, Yating Zhao, Xuan Zhu

**Affiliations:** College of Food Science and Pharmacy, Xinjiang Agricultural University, Urumqi 830052, China; 15776542799@163.com (X.Z.); 1869922855@163.com (Y.F.); zhaoyt@xjau.edu.cn (Y.Z.)

**Keywords:** non-thermal decontamination, pathogenic fungus, European plum, cell membrane integrity, antioxidant system

## Abstract

*Alternaria* sp. is a major pathogen causing postharvest storage decay of European plum. This study evaluated the antifungal activity of low-temperature plasma (LTP) against *Alternaria* sp. and elucidated its mechanism. With increasing LTP exposure, both mycelial growth and spore germination of *Alternaria* sp. were progressively inhibited; at 70 kV for 40 s, inhibition reached 100% and 99.32 ± 0.59%, respectively. Ultrastructural observations revealed abnormal spore morphology (swelling and shrinkage). Fluorescence microscopy showed compromised membrane integrity and increased permeability. The activities of catalase (CAT), superoxide dismutase (SOD) and peroxidase (POD) decreased by 86.08%, 82.86% and 75.47%, respectively, compared with the 0 s control group. Therefore, the accumulation of H_2_O_2_ and O_2_^−^ in cells destroys the redox homeostasis, further damages the membrane structure and function, and leads to the time-dependent increase in nucleic acid leakage, soluble protein leakage and malondialdehyde (MDA) accumulation. Collectively, LTP exhibits strong antifungal activity against *Alternaria* sp. and shows promise for controlling postharvest diseases of plum fruit.

## 1. Introduction

European plum (*Prunus domestica* L.) is native to Asia and is a climacteric fruit [1,2]. European plum fruit postharvest undergo rapid physiological and metabolic changes, and the fruit is highly vulnerable to phytopathogen infection during storage and transportation, with severe decay often induced as a consequence.

Black spot disease, predominantly caused by *Alternaria* sp., is recognized as one of the most destructive postharvest diseases of European plum, frequently resulting in substantial economic losses [3,4]. This necrotrophic fungus invades fruit tissues through wounds or natural openings, leading to characteristic dark lesions, tissue softening, and eventual fruit decay.

Currently, synthetic chemical fungicides remain the primary approach for managing postharvest fungal diseases. Nevertheless, mounting concerns over fungicide resistance, chemical residues in food products, and detrimental effects on environmental and human health have prompted increasingly stringent regulatory restrictions. Consequently, there is an urgent need to develop effective, safe, and environmentally sustainable decontamination alternatives for postharvest preservation.

Low-temperature plasma (LTP) has emerged as a promising non-thermal physical decontamination technology. Unlike conventional thermal or chemical methods, LTP generates a cocktail of reactive oxygen and nitrogen species (RONS), charged particles, and ultraviolet radiation at near-ambient temperatures, enabling potent antimicrobial activity without compromising food quality [5,6,7]. Although LTP has demonstrated efficacy against various foodborne pathogens and been widely applied to control *Aspergillus niger* on dates [8], mango anthracnose caused by *Colletotrichum* spp. [9], *Alicyclobacillus* spp. in apple juice [10], *Bacillus cereus* in red pepper powder [11], and *Aspergillus flavus* in peanuts [12], as well as *Escherichia coli* [13], *Salmonella* spp. [14], *Staphylococcus aureus* and *Botrytis cinerea* [15,16], and has been reported to inactivate fungi mainly by compromising plasma-membrane integrity and causing intracellular contents leakage [17], its specific effects and underlying antifungal mechanisms against *Alternaria* sp.—the primary causal agent of plum black spot and the major postharvest pathogen of European plum—remain poorly and insufficiently characterized.

In this study, the effects of LTP on *Alternaria* sp. were systematically investigated by measuring mycelial growth, mycelial dry weight, spore germination and morphology, antioxidant defense system, nucleic acid leakage, extracellular protein content and malondialdehyde (MDA) content. In order to clarify its pathogenicity changes, we further determined the incidence and lesion diameter of LTP-treated European plum fruits inoculated with spore suspension. The results provide a theoretical basis for the application of LTP in fruit postharvest preservation and expand its application potential in the food industry.

## 2. Materials and Methods

### 2.1. Fungi Strains and Culture Conditions

The fungal pathogen *Alternaria* sp. was isolated from naturally infected plum fruit and identified via ITS sequence analysis, GenBank accession No. PZ498049. The isolate was routinely cultured on potato dextrose agar (PDA) (Wuhan Servicebio Technology Co., Ltd., Wuhan, China) at 28 ± 1 °C and 80% relative humidity (RH) in the dark for 7 days.

### 2.2. Spore Suspensions Preparation and LTP Treatment

Spores on the plate surface were gently harvested by flooding with sterile phosphate-buffered saline (PBS) (Wuhan Servicebio Technology Co., Ltd., Wuhan, China). The resulting spore suspension was counted with a hemocytometer (YA0810-1EA; Beijing Solabao Technology Co., Ltd., Beijing, China) and adjusted by dilution to a final concentration of 1 × 10^7^ spores/mL.

As shown in Figure S1, the LTP setup consists of a power supply (CTP-2000K), a voltage regulator, and a dielectric barrier discharge (DBD) reactor (DBD-50; Nanjing Suman Plasma Technology Co., Ltd., Nanjing, China). An atmospheric-pressure LTP reactor (DBD-100A; Nanjing Suman Plasma Technology Co., Ltd., Nanjing, China; outer diameter 94 mm, inner diameter 61 mm, depth 8 mm) was placed on the low-voltage (grounded) electrode. The quartz reactor was covered with a quartz dielectric plate (diameter, 102 mm; thickness, 1 mm). The prepared *Alternaria* sp. spore suspension was placed in the reactor. The discharge gap (the distance between the sample and the high voltage electrode) is set to 8 mm, the discharge frequency is set to 1.5 kHz, and the working gas is ambient air at normal temperature and pressure. The voltage was adjusted to 70 k V, and the treatment time was 0, 10, 20, 40, and 80 s (the 0 s treatment group was designated as the untreated control). The temperature of spore suspension was always kept below 35 °C.

As shown in Figure S2, European plum fruits inoculated with *Alternaria* sp. spores were placed in a reactor. The discharge gap (the distance between the sample and the high-voltage electrode) was set to 5 mm, and the working gas was ambient air at normal temperature and atmospheric pressure. The applied voltage was adjusted to 70 kV, and treatment durations were 0, 10, 20, 40, and 80 s.

### 2.3. Measurement of Mycelial Growth of Alternaria sp.

The in vitro mycelial growth was assessed using the method described by [18]. Spore suspensions (1 × 10^7^ spores/mL), which had been pretreated with the LTP device for 0, 10, 20, 40, and 80 s, were dispensed into individual sterile containers, with the 0 s treatment group serving as the control. Subsequently, 10 μL aliquots of each spore suspension were inoculated onto the center of potato dextrose agar plates, and the plates were then incubated for 14 d in a constant temperature and humidity incubator. Colony diameters in each Petri dish were measured via the criss-cross method. Three independent biological replicates were performed.

### 2.4. Assay of Spore Germination of Alternaria sp.

Spore germination was evaluated using the spread plate method, as described by Li et al. [19]. Spore suspension (1 × 10^7^ spores/mL) of *Alternaria* sp. was evenly spread onto water agar plates, which were then incubated at 28 °C in a constant temperature incubator (SPX-150B-Z11 Biochemical Incubator Shanghai Boxun Medical Biological Instrument Co., Ltd. Shanghai, China). After 6 h, spore germination was observed under a light microscope (S550T-720HD Biological Microscope; Suzhou Beitejia Optoelectronics Technology Co., Ltd., Suzhou, China). The germination status of 100 spores per plate was recorded, and the germination rate was calculated following the method reported by Li et al. [19]. Three independent biological replicates were performed.

### 2.5. Dry Weight of Mycelia of Alternaria sp.

For mycelial dry weight estimation, the method described by Wang et al. [20] was adapted. Potato glucose agar liquid medium (70 mL) was added to an Erlenmeyer flask and sterilized using an autoclave (DGS-280B+ portable pressure steam sterilizer Jiangsu Dengguan Medical Instrument Co., Ltd., Changzhou, China). Each flask was inoculated with 1 mL of an *Alternaria* sp. spore suspension (1 × 10^7^ spores/mL), sealed with sterile Parafilm secured by a rubber band, and incubated at 28 °C for 7 d in a constant-temperature shaking incubator. Mycelial growth was harvested by filtration using a Büchner funnel, and the collected mycelia were dried in an oven at 75 °C until a constant weight was achieved. The mycelial dry weight (in grams) was subsequently measured. Three independent biological replicates were performed.

### 2.6. Observation of Microscopic Morphology of Alternaria sp.

The method described by Zhao et al. [21] was followed with modifications. After LTP treatment, spores of *Alternaria* sp. at 0 s and 80 s were fixed in electron microscopy fixative (24 h, 4 °C), followed by three washes with PBS. They were then gradient-eluted using ethanol solutions of increasing concentrations, with each step lasting 15 min. Afterward, the samples were dried using a critical point dryer (K850; Quorum., North Tyneside, UK). Following 30 s of gold sputter coating, the spore morphology was examined under the SEM (SU8100; HITACHI., Tokyo, Japan).

### 2.7. Measurement of Membrane Permeability of Alternaria sp.

The method described by Xu et al. [22] was used with slight modifications. A 1 mL aliquot of the spore suspension (1 × 10^7^ spores/mL) was transferred to a sterilized centrifuge tube and centrifuged at 8000× *g* for 10 min. The supernatant was discarded, and 500 μL of propidium iodide (PI) staining solution (10 μg/mL) was added to the spore pellet. After vortexing to ensure thorough mixing, the sample was incubated for 35 min in a 30 °C water bath in the dark, followed by centrifugation. The spore pellet was washed repeatedly with 500 μL PBS, with centrifugation after each wash to remove residual staining solution. Finally, the stained spores were resuspended in 600 μL of PBS and vortexed to ensure uniformity. Spore morphology was observed and imaged using a fluorescence microscope (DM3000-LED; Leica., Wetzlar, Germany). Three independent biological replicates were performed.

### 2.8. The Effect of LTP on the Antioxidant System of Alternaria sp.

The activities of catalase (CAT), superoxide dismutase (SOD), and peroxidase (POD), as well as the H_2_O_2_ and superoxide anion (O_2_^−^) contents in *Alternaria* sp. mycelia, were measured using biological kits (Beijing Solarbio Science & Technology Co., Ltd.,Beijing, China). The measurements were conducted at wavelengths of 405 nm, 450 nm, 470 nm, 405 nm, and 530 nm, respectively. Three independent biological replicates were performed.

### 2.9. Determination of Cell Component Release of Alternaria sp.

A modified protocol based on Ott et al. [23] was followed, where spore suspensions (1 × 10^7^ spores/mL) were treated with LTP treatment for 0, 10, 20, 40, and 80 s, with the 0 s treatment serving as the control. For both the treated and control groups, 200 μL of spore suspension was transferred into 50 mL Erlenmeyer flasks containing 10 mL of PDA liquid medium. The flasks were incubated at 28 °C for 1 h in a biochemical incubator (SPX-150B-Z; Shanghai Boxun Medical Biological Instrument Co., Ltd., Shanghai, China). After incubation, 2 mL of the spore suspension was aspirated and centrifuged at 8000 *g* for 10 min. The supernatant was collected, and its absorbance at 260 nm was measured using a UV spectrophotometer. Three independent biological replicates were performed.

### 2.10. Detection of Malondialdehyde and Protein Content of Alternaria sp.

Cellular protein content and the concentration of malondialdehyde (MDA) in *Alternaria* sp. following LTP treatment was evaluated using a BCA protein and MDA assay kit (Beijing Solarbio Science & Technology Co., Ltd., Beijing, China), respectively [24]. Three independent biological replicates were performed.

### 2.11. Antifungal Effect of LTP on Plum Fruit Inoculated with Alternaria sp.

We referred to the methods described by Sun et al. and Qian et al. [25,26]. Before inoculation, the surface of plum fruits was gently wiped with 75% ethanol solution, and then pierced (one wound of 3 mm in diameter and depth) at the equatorial part of the plum fruits utilizing a sterile perforator. Afterward, 20 μL of spore suspension (1 × 10^7^ spores/mL) was inoculated. Inoculated samples were treated with LTP and preserved at 28 ± 2 °C and 90–95% relative humidity for 14 d. The diameter of the lesion was measured daily, and the disease was considered to have developed when the diameter was more than 3 mm. Three independent biological replicates were performed, with 15 fruits per treatment group in each replicate (*n* = 15 per replicate, totaling 45 fruits per treatment group). Fruits were randomly allocated to treatment groups to minimize batch effects.

### 2.12. Statistical Analysis

All experiments were performed with three independent biological replicates. Data are expressed as mean ± standard deviation (SD). For assays involving a single time-point comparison across multiple treatment groups, one-way analysis of variance (ANOVA) was performed, followed by Duncan’s multiple range test was applied in SPSS 22.0 for statistical comparison, with *p* < 0.05 considered significant. Figures were drawn using Origin 2024.

## 3. Results

### 3.1. Effect of LTP on Mycelial Growth of Alternaria sp.

**Figure 1:**
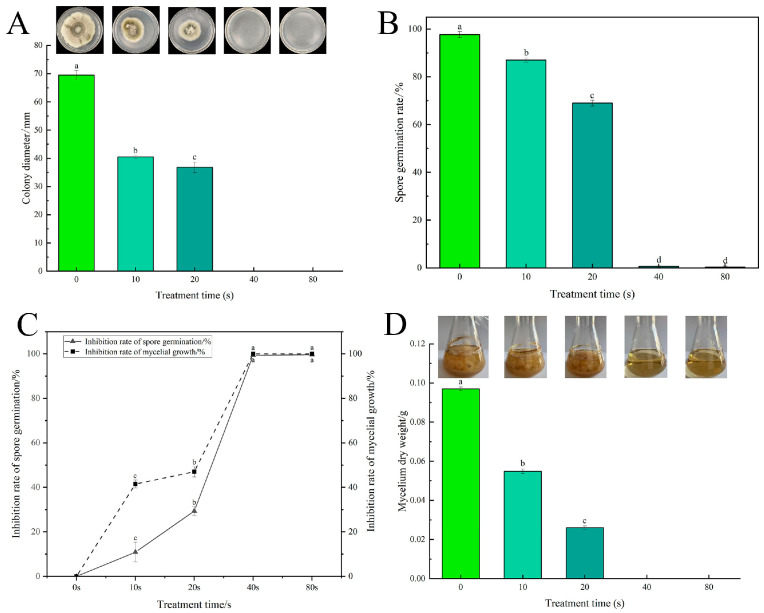
Effects of LTP on the colony diameter (**A**), spore germination rate (**B**), mycelial growth and spore germination inhibition rates (**C**), and biomass (**D**) of *Alternaria* sp. observed under an optical microscope. Vertical bars indicate standard errors of the means. Different letters (a–c) indicate statistically significant differences (*p* < 0.05).

Mycelia germinating from *Alternaria* sp. spores gradually developed into colonies over the incubation period. As shown in Figure 1A, LTP treatment markedly inhibited *Alternaria* sp. mycelial growth after 14 d of incubation at 28 °C. Significant differences in colony diameter were observed among treatments with different discharge durations (*p* < 0.05). The antifungal effect intensified with longer treatment times, accompanied by a progressive reduction in colony diameter. The colony diameter of the control group was 69.51 mm, which was significantly greater than that of all LTP-treated groups (*p* < 0.05). The 10 s and 20 s treatment groups had colony diameters of 40.67 mm and 36.83 mm, respectively. When the treatment time increased to 40 s, mycelial growth was completely inhibited, with no visible colonies on the Petri dishes.

### 3.2. Effect of LTP on Spore Germination of Alternaria sp.

As shown in Figure 1B, after 10, 20, 40, and 80 s of LTP treatment followed by 6 h of incubation, the germination rates of *Alternaria* sp. spores were 87.00%, 69.00%, 0.67%, and 0.33%, respectively—all significantly lower than that of the control 97.67 (*p* < 0.05). Significant differences were observed between each LTP-treated group and the control (*p* < 0.05).

As shown in Figure 1C, both the mycelial growth inhibition rate and spore germination inhibition rate of *Alternaria* sp. increased with longer LTP exposure. For treatment durations of 10, 20, 40, and 80 s, the mycelial growth inhibition rates were 41.48%, 47.00%, 79.43%, and 100%, respectively. Complete inhibition of mycelial growth occurred at 40 s and 80 s. The corresponding spore germination inhibition rates were 10.88%, 29.33%, 99.32%, and 99.66%, respectively. At treatment durations of 40 s and 80 s, spore germination was nearly eliminated, indicating a pronounced antifungal effect (*p* < 0.05).

### 3.3. Effect of LTP on Dry Weight of Alternaria sp. Mycelia

The inhibitory effect of LTP on *Alternaria* sp. mycelial biosynthesis was evaluated by measuring the dry weight of the mycelia [27]. As shown in Figure 1D, the mycelial biomass of *Alternaria* sp. gradually decreased with increasing treatment duration. The biomass in the control group was 0.097 g. After 10 s and 20 s of treatment, the mycelial biomass decreased to 0.055 g and 0.026 g, representing reductions of 43.3% and 73.2% compared with the control (*p* < 0.05). When the treatment duration was extended to 40 s and 80 s, the mycelial biomass was 0 g, indicating complete inhibition of mycelial growth.

### 3.4. Effect on Cell Morphology of Alternaria sp.

**Figure 2:**
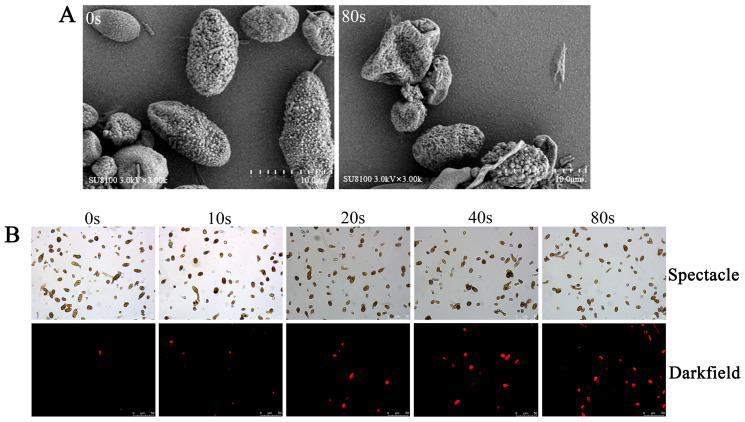
Effects of LTP treatment on the surface morphology and cell membrane integrity of *Alternaria* sp. spores. (**A**) Scanning electron microscopy images showing the surface morphology of control and 80 s LTP-treated spores. (**B**) Fluorescence microscopy images showing the effects of LTP on spore membrane integrity, with Spectacle and Darkfield representing observations under light and dark conditions, respectively.

As shown in Figure 2A, spores of *Alternaria* sp. in the control group exhibited intact, plump, and smooth surfaces. After treatment with the LTP device at 70 kV for 80 s, the surface morphology of the spores changed markedly compared with the control, showing indentations and shriveling.

### 3.5. Effect of LTP on Membrane Permeability of Alternaria sp.

PI staining is commonly used to assess membrane integrity [28]. As shown in Figure 2B, following 10 s of LTP treatment, partial red fluorescence was observed under dark-field microscopy. As the treatment duration increased to 20, 40, and 80 s, the intensity and extent of red fluorescence gradually increased. In contrast, almost no red fluorescence was observed in the control group, indicating that LTP disrupted the integrity of *Alternaria* sp. spore membranes, allowing PI to enter the cells. The longer the LTP exposure, the more severe the damage to the cell membranes of *Alternaria* sp. spores.

### 3.6. Effect of LTP on O_2_^−^ and H_2_O_2_ Content of Alternaria sp.

**Figure 3:**
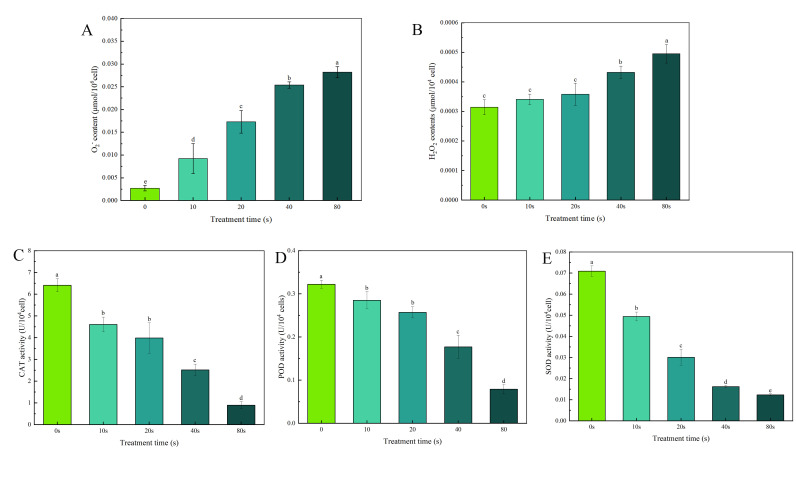
Effects of LTP treatment on the antioxidant system of *Alternaria* sp. (**A**,**B**) Levels of O_2_^−^ and H_2_O_2_ in *Alternaria* sp. spores after LTP treatment. (**C**–**E**) Activities of CAT, POD, and SOD in *Alternaria* sp. spores following LTP treatment. Vertical bars indicate the standard error of the mean. Different letters (a–e) indicate statistically significant differences (*p* < 0.05).

High concentrations of O_2_^−^ and H_2_O_2_ can disrupt fungal cell membranes, leading to cellular necrosis, senescence, and eventual lysis [29]. As shown in Figure 3A, the intracellular O_2_^−^ content in the 10, 20, 40, and 80 s LTP-treated groups was significantly higher than that in the 0 s control group (*p* < 0.05). From 0 to 80 s, intracellular O_2_^−^ levels increased progressively with treatment duration.

As presented in Figure 3B, H_2_O_2_ content increased from 0.00031 μmol/10^4^ cell (0 s) to 0.00034 μmol/10^4^ cell (10 s), 0.00035 μmol/10^4^ cell (20 s), 0.00043 μmol/10^4^ cell (40 s), and 0.00049 μmol/10^4^ cell (80 s). Compared with the control (0 s), H_2_O_2_ content increased by 9.677%, 12.903%, 38.710%, and 58.065%, respectively.

### 3.7. Effect of LTP on CAT, POD, and SOD Activities of Alternaria sp.

According to Figure 3C, CAT activity declined from 6.410 U/10^4^ cell (0 s) to 4.608 U/10^4^ cell (10 s), 3.985 U/10^4^ cell (20 s), 2.516 U/10^4^ cell (40 s), and 0.892 U/10^4^ cell (80 s), corresponding to reductions of 28.111%, 37.831%, 60.749%, and 86.084%, respectively, relative to the control.

Similarly, as shown in Figure 3D, POD activity decreased from 0.322 U/10^4^ cell (0 s) to 0.285 U/10^4^ cell (10 s), 0.257 U/10^4^ cell (20 s), 0.177 U/10^4^ cell (40 s), and 0.079 U/10^4^ cell (80 s), representing declines of 11.490%, 20.186%, 45.031%, and 75.466%, respectively. The activities of CAT and POD exhibited a consistent downward trend with increasing LTP exposure, inversely correlated with H_2_O_2_ accumulation. These findings indicate that decreased antioxidant enzyme activity contributes to the intracellular buildup of H_2_O_2_.

As shown in Figure 3E, the effects of different LTP treatment durations on *Alternaria* sp. SOD activity were examined. A 10 s LTP treatment caused only a slight change in SOD activity. After 20, 40, and 80 s of exposure, SOD activity decreased to 0.049 U/10^4^ cell, 0.030 U/10^4^ cell, and 0.012 U/10^4^ cell, respectively—all significantly lower than the 0 s control 0.070 U/10^4^ cell (*p* < 0.05). These results indicate that LTP treatment suppresses SOD activity in *Alternaria* sp., with the inhibitory effect becoming more pronounced as treatment duration increases.

### 3.8. Effect of LTP on the Release of Cellular Components and MDA Content from Alternaria sp. Spores

**Figure 4:**
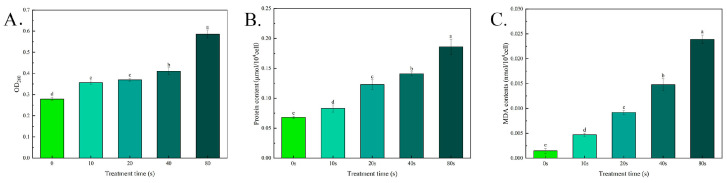
Levels of extracellular nucleotide leakage, protein content, and MDA content. Vertical bars represent the standard error of the mean. (**A**) Extracellular nucleic acid leakage, (**B**) Extracellular protein concentration, (**C**) Extracellular malondialdehyde content. Different letters (a–e) indicate statistically significant differences (*p* < 0.05).

The integrity of the cell membrane was indirectly evaluated by monitoring the release of intracellular nucleic acids. When membrane integrity is compromised, intracellular components such as DNA and RNA leak into the extracellular environment. This leakage leads to an increase in absorbance at 260 nm (OD_260_), which is positively correlated with the concentration of extracellular nucleic acids. Therefore, OD_260_ measurements can serve as an indirect indicator of membrane damage [30]. As shown in Figure 4A, LTP treatment significantly increased the release of cellular constituents from *Alternaria* sp. After 10 s of treatment, the OD_260_ absorbance rose markedly compared with the control group. The OD_260_ values for the 10, 20, 40, and 80 s treatment groups were 0.357, 0.370, 0.410, and 0.586, respectively—all significantly higher than that of the control 0.279 (*p* < 0.05).

The leakage of Intracellular proteins is also a key indicator for assessing whether the cell membrane is intact. As shown in Figure 4B, with increasing LTP treatment duration (from 0 s to 80 s), the protein concentration in the extracellular supernatant increased significantly from 0.068 μmol/10^4^ cell to 0.186 μmol/10^4^ cell (*p* < 0.05). Membrane integrity was further evaluated by quantifying malondialdehyde (MDA). As shown in Figure 4C, the MDA concentration increased significantly with treatment duration, rising from 0.0015 nmol/10^4^ cell at 0 s to 0.0239 nmol/10^4^ cell at 80 s (*p* < 0.05).

### 3.9. Antifungal Effect of LTP on Disease Development in Plum Fruit

**Figure 5:**
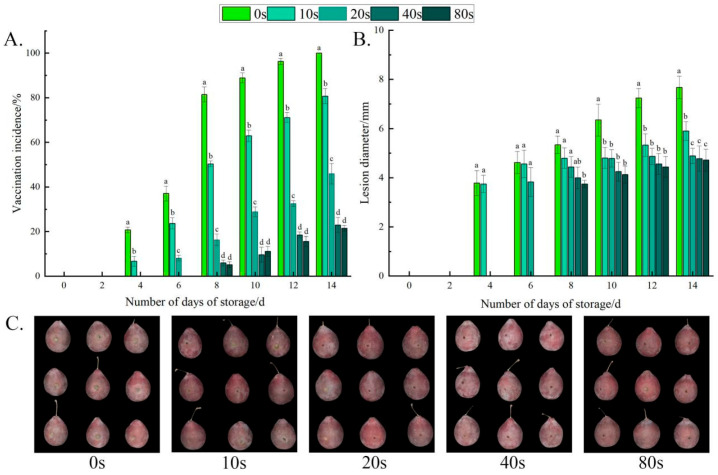
Effects of LTP treatment on the incidence of black spot disease (**A**) and lesion diameter (**B**) in plum fruits after 14 days of storage, as well as the corresponding disease symptoms on day 14 (**C**). Vertical bars represent the standard error of the mean. Different letters (a–d) indicate statistically significant differences (*p* < 0.05).

As shown in Figure 5A, disease symptoms appeared in the control group of plum fruits on the ^4t^h day of storage, four days earlier than in the 80 s treatment group. By day 6, fruits in the control, 10 s, 20 s, and 40 s treatment groups exhibited varying degrees of infection, whereas no visible symptoms were observed in the 80 s group. On day 14, the disease incidence in the control group reached 100%, while the incidences in the 10 s, 20 s, 40 s, and 80 s treatment groups were 80.74%, 45.93%, 22.96%, and 21.48%, respectively, representing significant reductions of 19.26%, 54.07%, 77.04%, and 78.52% compared with the control (*p* < 0.05).

As shown in Figure 5B, from day 8 onward, the lesion diameters of plum fruits in the 40 s and 80 s treatment groups were significantly smaller than those of the control. At the end of storage, the average lesion diameter in the control group was 7.68 mm, while those in the 10 s, 20 s, 40 s, and 80 s groups were 5.90 mm, 4.89 mm, 4.78 mm, and 4.78 mm, corresponding to reductions of 23.18%, 36.33%, 37.76%, and 38.54%, respectively (*p* < 0.05).

## 4. Discussion

LTP is recognized as a safer and more effective alternative to chemical fungicides and has been increasingly applied for the microbial decontamination of agricultural produce [8,31]. In this study, we systematically evaluated the inhibitory effects of LTP on *Alternaria* sp. by examining mycelial growth, spore germination, biomass accumulation, spore ultrastructure, and the fungal antioxidant system, and also determined the impact of LTP on the pathogenicity of *Alternaria* sp.

Our results demonstrated that LTP treatment effectively inhibited *Alternaria* sp., with complete suppression of mycelial growth and spore germination after 40 s of exposure. SEM analysis revealed significant morphological abnormalities in treated spores, including pronounced shrinkage and collapse. Consistent with these findings, Cao et al. [32] reported that LTP caused shrinkage, deformation, and collapse in Botrytis cinerea spores—effects associated with inhibited mycelial growth and spore germination [33]. PI, a red fluorescent nucleic acid stain that can only enter cells with damaged membranes, binds to DNA and emits red fluorescence under a fluorescence microscope. Therefore, PI staining is commonly used to assess cell membrane integrity [34]. In our fluorescence microscopy analysis, red fluorescence intensity increased progressively with longer LTP exposure compared with the 0 s control, indicating that LTP treatment compromised cell membrane integrity, leading to a loss of cell viability [35].

LTP effectively inactivates *Alternaria* sp. not only by directly damaging the cell membrane, altering cell morphology, and disrupting membrane integrity and permeability, but also by impairing intracellular redox homeostasis. This impairment leads to the accumulation of intracellular O_2_^−^ and H_2_O_2_, thereby inducing lipid peroxidation [25]. Previous studies have demonstrated that changes in intracellular O_2_^−^ and H_2_O_2_ levels can regulate secondary metabolism [36]. When the accumulation of O_2_^−^ and H_2_O_2_ exceeds the scavenging capacity of the antioxidant defense system, high levels of these reactive oxygen species (ROS) cause oxidative stress and cellular damage [37,38], ultimately resulting in impairment of the membrane structure of *Alternaria* sp. Under normal physiological conditions, cells produce various antioxidant enzymes such as SOD, POD, and CAT, which scavenge excess O_2_^−^ and H_2_O_2_ to protect cells from oxidative stress [39]. Specifically, both CAT and POD can decompose excess H_2_O_2_ [40], while SOD eliminates intracellular O_2_^−^ accumulation [41]. In the present study, LTP treatment significantly reduced the activities of SOD, POD, and CAT, which was accompanied by significantly higher H_2_O_2_ and O_2_^−^ contents in all treatment groups compared with the 0 s control group. These observations are consistent with a model in which impaired antioxidant capacity contributes to oxidative stress and subsequent membrane damage in *Alternaria* sp. It should be noted, however, that the enzyme activity declines measured in bulk lysates may partly reflect non-specific protein inactivation rather than selective inhibition of these enzymes [42].

Nucleic acid leakage and the concentrations of extracellular proteins and MDA are key indicators of plasma membrane integrity. In *Alternaria* sp. spores, LTP treatment caused a time-dependent increase in nucleic acid leakage, extracellular protein concentration, and MDA content. Consistent with these results, Wei et al. [43] observed increased nucleic acid leakage in Aspergillus niger spores following LTP treatment, while Sun et al. [25] reported elevated extracellular protein and MDA levels in Fusarium verticillioides spores, and Du et al. [44] observed similar membrane damage indicators in Staphylococcus aureus under plasma treatment. The leakage of intracellular components impairs essential physiological and biochemical processes, inhibits fungal growth, and may ultimately lead to cell death.

The impact of LTP on *Alternaria* sp. pathogenicity was evaluated using infection assays. After 14 d of storage, severe decay was observed in the control group, whereas disease incidence was significantly reduced in the 80 s LTP-treated group. These findings indicate that LTP treatment effectively inhibits *Alternaria* sp. mycelial growth and spore germination, thereby reducing its pathogenicity and the incidence of black spot on plums. In agreement with these results, Pereira et al. [45] also reported that LTP treatment markedly reduced the pathogenicity of *Alternaria* sp. and decreased the incidence of brown rot in apples.

**Figure 6:**
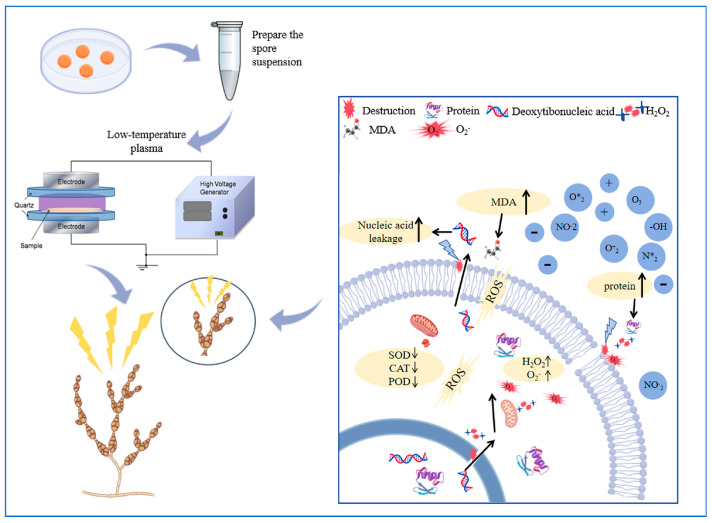
Antifungal mechanism of low-temperature plasma.

Collectively, our data establish that LTP exerts antifungal activity against *Alternaria* sp. through a multi-target oxidative mechanism encompassing membrane disruption and antioxidant system inactivation (Figure 6). This dual action renders the fungus unable to mount effective stress responses, leading to irreversible oxidative damage and metabolic dysfunction.

## 5. Conclusions

In conclusion, this study set out to evaluate the efficacy of LTP against *Alternaria* sp. and to elucidate its underlying physiological mechanisms. Our findings demonstrate that LTP treatment exerts a dose-dependent inhibitory effect on fungal growth and pathogenicity. Mechanistically, our findings suggest that this antifungal activity is associated with disruption of cellular membrane integrity and suppression of the enzymatic antioxidant defense system, which may collectively contribute to oxidative damage and metabolic dysfunction in the fungal cells. These results confirm that LTP operates through a multi-target oxidative pathway to deactivate *Alternaria* sp. While this work provides a foundational understanding of the oxidative stress responses, further investigations are warranted to decipher the interactions between plasma-generated reactive species and intracellular genetic material. Given its notable efficiency and low environmental impact, LTP shows considerable promise as a viable alternative for managing fungal contaminants in agricultural or food systems.

## Data Availability

The original contributions presented in this study are included in the article/Supplementary Material. Further inquiries can be directed to the corresponding author.

## Supplementary Materials

The following supporting information can be downloaded at: https://www.mdpi.com/article/10.3390/foods15152702/s1, Figure S1: SLTP apparatus designed for the treatment of Alternaria sp. spore suspensions.; Figure S2: LTP apparatus designed for the treatment of European plum fruit.
