# Supplementary material for: Low Temperature Plasma Impairs the Pathogenicity of Alternaria sp. by Disrupting Its Cell Membrane and Redox Homeostasis
Source: Foods. 2026 Jul 31;15(15):2702. doi: 10.3390/foods15152702 (PMC13463994; doi:10.3390/foods15152702)
Supplement: Supplementary file 1 [file foods-15-02702-s001.zip › foods-4404339-supplementary.pdf]

foods

MDPI

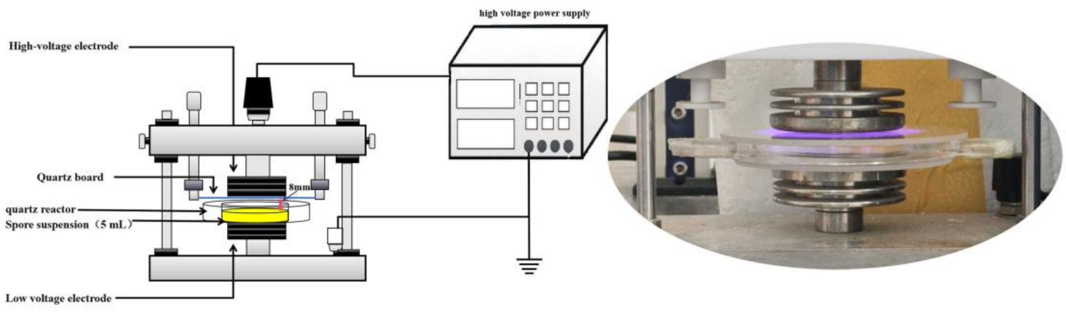

Figure S1. LTP apparatus designed for the treatment of *Alternaria sp.* spore suspensions.

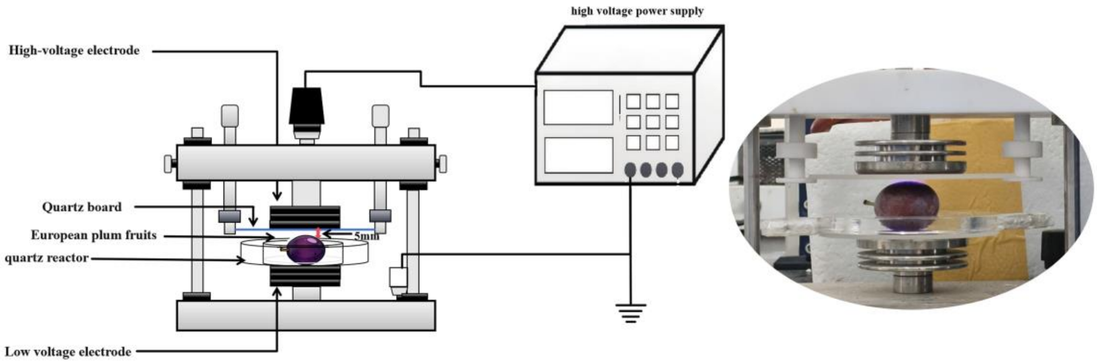

Figure S2. LTP apparatus designed for the treatment of European plum fruit.
